# Supplementary material for: MetaRibo-Seq measures translation in microbiomes
Source: Nat Commun. 2020 Jun 29;11:3268. doi: 10.1038/s41467-020-17081-z (PMC7324362; doi:10.1038/s41467-020-17081-z)
Supplement: Supplementary file 10 — Supplementary Data 7 [file 41467_2020_17081_MOESM10_ESM.zip › File2/Confidence_High_Taxonomy/373167_out.krona.html]

Javascript must be enabled to view this page.

members
magnitude
magnitudeUnassigned
count
unassigned
taxon
rank

373167\_out

3

superkingdom
2
3

phylum
976
3

class
200643
3

order
3
171549

family
3
171552

genus
577309
3

species
454154
3

SRS053356\_contig\_number\_23521SRS1041031\_contig\_number\_26920SRS1041140\_contig\_number\_8476
